# Unemployment and Health-Related Quality of Life in Melanoma Patients During the COVID-19 Pandemic

**DOI:** 10.3389/fpubh.2021.630620

**Published:** 2021-02-22

**Authors:** Yeye Guo, Minxue Shen, Xu Zhang, Yi Xiao, Shuang Zhao, Mingzhu Yin, Wenbo Bu, Yan Wang, Xiang Chen, Juan Su

**Affiliations:** ^1^Department of Dermatology, Xiangya Hospital, Central South University, Changsha, China; ^2^National Clinical Research Center for Geriatric Disorders, Xiangya Hospital, Changsha, China; ^3^Hunan Engineering Research Center of Skin Health and Disease, Changsha, China; ^4^Hunan Key Laboratory of Skin Cancer and Psoriasis, Xiangya Hospital, Central South University, Changsha, China; ^5^Department of Social Medicine and Health Management, Xiangya School of Public Health, Central South University, Changsha, China; ^6^Jiangsu Key Laboratory of Molecular Biology for Skin Diseases and STIs, Institute of Dermatology, Chinese Academy of Medical Science & Peking Union Medical College, Nanjing, China

**Keywords:** melanoma, coronavirus disease 2019, unemployment, quality of life, income loss

## Abstract

The outbreak of coronavirus disease-2019 (COVID-19) ineluctably caused social distancing and unemployment, which may bring additional health risks for patients with cancer. To investigate the association of the pandemic-related impacts with the health-related quality of life (HRQoL) among patients with melanoma during the COVID-19 pandemic, we conducted a cross-sectional study among Chinese patients with melanoma. A self-administered online questionnaire was distributed to melanoma patients through social media. Demographic and clinical data, and pandemic-related impacts (unemployment and income loss) were collected. HRQoL was determined by the Functional Assessment of Cancer Therapy-General (FACT-G) and its disease-specific module (the melanoma subscale, MS). A total of 135 patients with melanoma completed the study. The mean age of the patients was 55.8 ± 14.2 years, 48.1% (65/135) were male, and 17.04% (34/135) were unemployed since the epidemic. Unemployment of the patients and their family members and income loss were significantly associated with a lower FACT-G score, while the MS score was associated with the unemployment of the patients' family members. Our findings suggested that unemployment is associated with impaired HRQoL in melanoma patients during the COVID-19 epidemic.

## Introduction

The outbreak and pandemic of the coronavirus disease 2019 (COVID-19) brought profound impacts on the entire society and the individual's life. Besides the confirmed and suspected cases in hospitals or health facilities, the majority of people started the self-isolation at home voluntarily. However, the lockdown resulted in income loss and unemployment in some people. Under the circumstances, patients with cancers or chronic conditions may face higher risks of job loss and more mental and physical stress.

Melanoma is the most serious type of skin cancers with a highly aggressive ability. The 5-years survival rate can be low as 5% for advanced melanoma (1), and the median overall survival time is about 8 months (2). Such poor survival outcomes result in heavy mental stress and impaired quality of life. Approximately one-third of patients with melanoma reported psychological distress to some extents, and symptoms of anxiety was more prevalent than depression (3). Neoadjuvant therapy such as checkpoint therapy and target therapy has been rapidly developed in recent years, and significantly benefited melanoma patients. However, high medical costs imposed heavy financial burdens on these patients. It was estimated that over 25,000 US dollars were cost per person annually for patients in late stages (4). As a result, unemployment and income loss may not only result in emotional problems but also lead to unaffordability during the advanced treatment for melanoma.

In the current study, we investigated the association of unemployment, income loss and other epidemic-related impacts with the health-related quality of life (HRQoL) in melanoma patients in China, based on an online questionnaire survey.

## Materials and Methods

### Study Design and Participants

This was a cross-sectional study among Chinese patients with melanoma. We created an online survey link to facilitate the collection of questionnaires, and distributed it on social media (WeChat groups and teledermatology platforms where melanoma patients could communicate with other patients and doctors). Every single IP address was allowed only one entry and submission in order to avoid repeated submissions by individual patients. The final submission required the patients to complete all the questions. A *post hoc* analysis for sample size were performed through SAS Macro. The means and standard deviations of FACT-G were 77.53 ± 15.39 in the unaffected group (*N* = 58) and 64.26 ± 18.15 in the unemployed group (*N* = 23). According to the method of sample size estimation for Satterthwaite *t*-test proposed by Moser (5), assuming a double-sided significance level of 0.05, a power of 80%, and a sample ratio of 2:1, the minimum sample sizes will be 44 and 22, respectively, for each group. The survey was conducted between 4 Apr, 2020 and 15 Jan, 2021. The study was reviewed and approved by the institutional research ethics boards of Xiangya Hospital, Central South University (approval number: 202002024). Electronic informed consent was collected from all participants before the survey.

### Exposure

The employment status was determined by a single question “What is your employment status since the epidemic of COVID-19?” with the following three responses: “I am unemployed since the epidemic,” “My employment status is unaffected since the epidemic,” and “I was unemployed or retired before the epidemic.” The employment status of the respondent's family members was also inquired in a similar way.

Income change was determined by a single question “Since the epidemic of COVID-19, is there any change in your monthly income?” with the following responses: “Complete income loss,” “Income reduced,” “Income unaffected (including the situation that I had no income before the epidemic),” and “income increased.” Because only one patient reported increased income, this patient was also categorized as the “unaffected” group in the analysis.

Outdoor activity restriction was measured by a single question “During the past 2 weeks, how has your outdoor activity been affected?” with the following four responses: “My outdoor activity was unaffected,” “My outdoor activity was partly restricted,” and “I was isolated at home and receiving medical observation,” “I was quarantined in hospital and receiving medical observation or treatment.” Those who were isolated at home or in hospital were combined as one group.

### Outcome

Functional Assessment of Cancer Therapy-Melanoma (FACT-M) was developed to evaluate the quality of life in melanoma patients (www.facit.org). FACT-M is an outcome measurement system composed of a core questionnaire for cancer patients, the FACT-General (FACT-G), and a disease-specific module (6). The questionnaire comprises of six subscales: physical well-being (PWB), social/family well-being (SWB), emotional well-being (EWB), functional well-being (FWB), melanoma subscale (MS), and melanoma surgery scale (MSS). The first four subscales belong to the FACT-G and the last two subscales are melanoma-specific elements. In our study, the primary outcome was the FACT-G score (the sum of PWB, SWB, EWB, and FWB subscales), and the secondary outcome was the MS score. The possible score ranges of the FACT-G and MS were 0–108 and 0–64, respectively, and higher scores indicate better HRQoL. The MSS was not analyzed since not all patients received surgery. The use of the FACT-M was authorized, and the Chinese version was obtained from the copyright holder before the survey.

### Co-variates

Demographic and clinical information including gender, age, educational level (primary school and below, middle school, high school, college, graduate school), annual income (Chinese yuan, CNY), marital status (unmarried, married, divorced, widowed), clinical stage of melanoma (I, II, III, IV), course of melanoma (<1 year, 1–2 years, ≥3 years, site of melanoma (extremities, trunk, head and neck, mucosal, unknown, other), current status of disease (stable, recurrent, metastasis), comorbidities (hypertension, diabetes, coronary heart disease, gout, etc.), adherence to treatment (no treatment prescribed, adherent to treatment, not adherent to treatment), and healthcare utilization (hospital visit, telemedicine) were collected and analyzed as covariates. For educational level, college and graduate school were combined as one group. Divorcement was not reported and therefore was not presented in the result. Because most patients had acral melanoma, all other sites were combined.

More details of the questionnaire are shown in Supplementary Data.

### Statistical Analyses

There was no missing data in the questionnaire survey. The data were exported from the online survey system and analyzed with R version 3.5.2. Continuous variables with normal distribution were expressed as mean ± standard deviation (SD) and compared with analysis of variance (ANOVA). Hotelling's T2 test was used for the multivariate comparison of multiple subscale scores. Continuous data with skewed distribution were presented as median (interquartile range, IQR) and compared with Wilcoxon rank sum test. Categorical variables were summarized as counts (percentages) and compared using the chi-square test or Fisher's exact test. Stepwise linear regression with the corrected Akaike information criteria was used to identify variables that were associated with the outcomes. The effect size of the association was presented as regression coefficient and 95% confidence interval. *P* < 0.05 was considered statistically significant. Reporting of the results followed the STROBE guideline.

## Results

From April 4, 2020 to Apr 11, 2020, a total of 135 valid questionnaires was collected and analyzed. None reported confirmed infection with COVID-19. The mean age of the patients was 55.8 ± 14.2 years, and 65 (48.1%) were male. The characteristics of participants by employment status were shown in Table 1. Educational level, marital status, history of hypertension, income change since the epidemic, and employment status of family members were significantly different across the groups. The intracluster correlation coefficients of the subscales of FACT-M varied from 0.82 to 0.92, indicating good internal reliability. The mean FACT-G and MS scores were 72.1 ± 17.1 and 52.5 ± 7.6, respectively.

**Table 1:** Demographic and clinical characteristics of the participants.

**Characteristics**	**Total** ** (*N =* 135)**	**Employment status**			***P***
		**Unemployed since the** ** epidemic (*N =* 23)**	**Unaffected since the** ** epidemic (*N =* 58)**	**Unemployed before the epidemic** ** or retired (*N =* 54)**	
Age (year), mean ± SD	55.8 ± 14.2	49.9 ± 10.7	50.6 ± 12.8	64.0 ± 14.0	<0.001
Sex, %					0.869
Male	48.1	43.5	50.0	48.2	
Female	51.9	56.5	50.0	51.8	
Educational level, %					<0.001
Primary school and below	19.3	13.0	10.3	31.5	
Middle school	33.3	60.9	32.8	22.2	
High school	20.0	21.7	12.1	27.8	
College and above	27.4	4.4	44.8	18.5	
Annual income (CNY), %					<0.001
<10,000	42.2	47.8	20.7	63.0	
10,000–49,999	31.9	43.5	34.5	24.1	
50,000–99,999	20.0	8.7	32.8	11.1	
100,00–199,000	4.4	0.0	8.6	1.9	
>200,000	1.5	0.0	3.4	0.0	
Marriage, %					0.018
Unmarried	3.7	8.7	1.7	3.7	
Married	89.6	91.3	96.6	81.5	
Widowed	6.7	0.0	1.7	14.8	
Stage of melanoma, %					0.289
I	27.4	34.8	29.3	22.2	
II	34.1	17.4	37.9	37.0	
III	24.4	26.1	25.9	22.2	
IV	14.1	21.7	6.9	18.5	
Course of melanoma (year), %					0.113
<1	33.3	30.4	29.3	38.9	
1–2	52.6	39.1	58.6	51.9	
≥3	14.1	30.4	12.1	9.3	
Site of melanoma, %					0.752
Extremities	86.7	82.6	86.2	88.9	
Other sites	13.3	17.4	13.8	11.1	
Current status of melanoma, %					0.0.024
Stable	71.9	56.5	86.2	63.0	
Recurrent	14.1	21.7	8.6	16.6	
Metastasis	14.1	21.7	5.2	20.4	
History of disease, %					
Hypertension	29.6	8.7	20.7	48.1	<0.001
Diabetes	20.7	17.4	15.5	27.8	0.253
Coronary heart disease	11.9	8.7	1.7	24.1	0.001
Outdoor activity restriction					0.007
Outdoor activity unrestricted	66.7	39.1	67.2	77.8	
Outdoor activity partly restricted	17.8	26.1	22.4	9.3	
Isolated at home or in hospital	15.6	34.8	10.3	13.0	
Income change since the epidemic, %					<0.001
Complete loss	17.7	39.1	8.6	18.5	
Reduced	39.3	56.5	44.8	25.9	
Unaffected	43.0	4.3	46.6	55.5	
Unemployment of family members, %					<0.001
Yes	33.3	73.9	19.0	31.5	
No	66.7	26.1	81.0	68.5	
Adherence to treatment, %					0.179
No treatment prescribed	48.1	30.4	53.4	50.0	
Not adherent to the treatment	23.7	34.8	15.5	27.8	
Adherent to the treatment	28.1	34.8	31.0	22.2	
Healthcare utilization					
Visited a doctor in hospital, %	31.1	34.8	27.6	33.3	0.738
Consulted a doctor remotely, %	28.9	30.4	27.6	29.6	0.956

**Figure 1:**
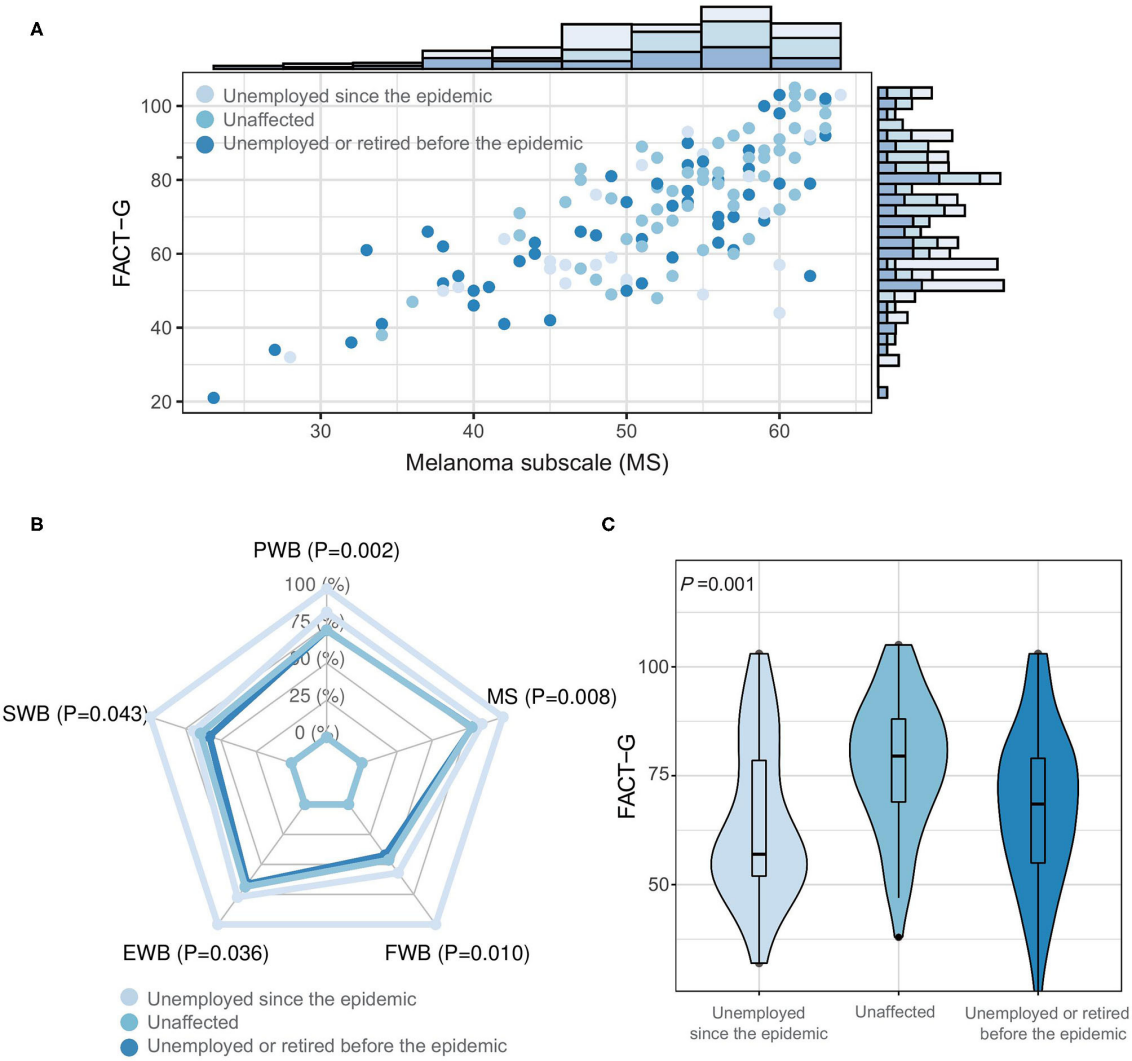
Association of unemployment with health-related quality of life in melanoma patients. **(A)** Scatter plot between FACT-G and MS scores by the employment status. **(B)** Mean subscale scores of FACT-G by the employment status. **(C)** Violin-boxplot for the distribution of FACT-G score by the employment status. FACT-G, Functional Assessment of Cancer Therapy-General; MS, melanoma subscale; PWB, physical well-being; SWB, social/family well-being; EWB, emotional well-being; FWB, functional well-being.

**Figure 2:**
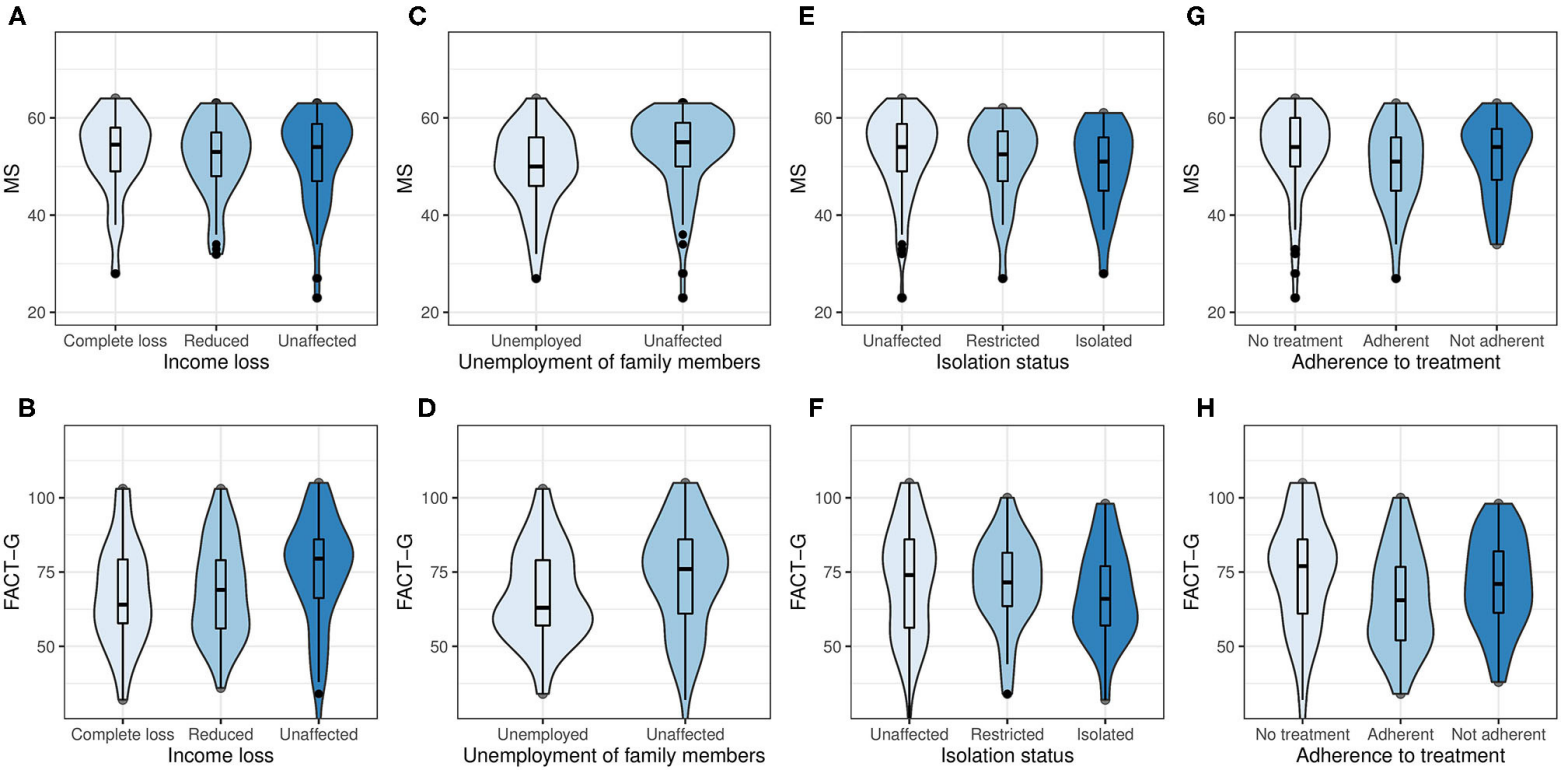
Violin-boxplot for the distribution of the FACT-G and MS scores by income loss **(A,B)**, unemployment of family members **(C,D)**, isolation status **(E,F)**, and adherence to treatment **(G,H)**.

The distribution of the FACT-G and MS scores by employment status are shown in Figure 1A, with a clear positive correlation between the two scores. According to the Hotelling's Trace for multivariate test, unemployment was significantly associated with the PWB, SWB, and FWB subscale scores, but was not associated with the EWB and MS subscale scores (Figure 1B). The FACT-G score was the lowest in patients who were unemployed since the epidemic (*P* = 0.001); in contrast, the difference was not significant between the patients who were unaffected and those who were unemployed or retired before the epidemic (Figure 1C). Income loss and unemployment of family members were also significantly associated with lower FACT-G but not MS score, while adherence to treatment and isolation status were not associated with both outcomes (Figure 2). It is noteworthy that the patients who were unemployment during the epidemic had a lower proportion of immune therapy at present (20.0%) compared to those whose income were unaffected (70.6%).

The stepwise linear regression identified lower educational level, unemployment since the epidemic, and progression of melanoma as risk factors for impaired HRQoL measured as the FACT-G (Table 2). The progression of melanoma and unemployment of family members were significantly associated with the MS score.

**Table 2:** Stepwise linear regression for the FACT-G and MS scores.

**Outcomes**	**Selected factors**	**Coefficient (SE)**	***P***
FACT-G	Education		
	Primary school and below	Reference	
	Middle school	4.9 (3.6)	0.173
	High school	14.6 (3.9)	<0.001
	College and above	14.0 (3.8)	<0.001
	Employment status		
	Unemployed since the epidemic	−14.9 (3.6)	<0.001
	Unaffected	Reference	
	Unemployed before the epidemic or retired	−3.3 (2.9)	0.259
	Current status of melanoma, n (%)		
	Stable	Reference	
	Recurrent	−10.9 (3.6)	0.003
	Metastasis	−24.8 (3.6)	<0.001
MS	Current status of melanoma, n (%)		
	Stable	Reference	
	Recurrent	−5.0 (1.7)	0.003
	Metastasis	−14.6 (1.7)	<0.001
	Unemployment of family members		
	No	Reference	
	Yes	−4.3 (1.2)	<0.001

*SE, standard error. FACT-G, Functional Assessment of Cancer Therapy-General, MS, melanoma subscale*.

## Discussion

In this study, we investigated the pandemic-related impacts on the HRQoL in melanoma patients through an online survey. The impaired HRQoL of melanoma was associated with the unemployment of the patients and their family members, as well as income loss. This is the first impact analysis of the COVID-19 for melanoma patients. These findings provide new insights into the health inequity issue arisen in the particular period.

Over 95 million people were documented to be infected with COVID-19 according to the World Health Organization (www.who.int). Besides the diseases, the pandemic led to a series social problems including reduced job opportunities. As reported by the United States Bureau of Labor Statistics, the unemployment rate was 12% in July 2020, which exhibited the profound influence of the pandemic on the social economy. In the current study, we found that 17.04% of the melanoma patients were unemployed since the epidemic of COVID-19 and their HRQoL were decreased notably. It is well-established that the health status and psychological characteristics of patients are associated with HRQoL. Previous studies found that patients with melanoma perceived persistent worries about developing new or metastatic cancers (7). A pervasive sense of uncertainty increases psychological distress, and impairs quality of life (8). Investigators found that systemic therapies can decrease HRQoL in a short time; however, the long-term HRQoL of melanoma survivors is comparable with the general population (9, 10). Interestingly, unemployment and a history of melanoma were previously identified to be associated with greater cancer worry, which indicated the importance of both melanoma and unemployment on psychological status (11). Our findings further emphasize the need to track the mental health in cancer patients under unemployment.

It has been estimated that over 100,000 people will be diagnosed with melanoma this year in the United States according to American Society of Clinical Oncology (ASCO). The incidence rate of melanoma in China was estimated at 0.9 per 100,000, with a 110.3% rise compared to the 1990s (12). The increasing incidence of melanoma makes it a public health concern and brings the global burden of disease (13). The disability-adjusted life-years (DALYs) of melanoma have been increasing in China during the last decade, while the DALYs of common cancers such as esophageal cancer and stomach cancer have decreased significantly (12). In recent years, the development of immune therapy benefits cancer patients substantially, but also leads to a heavy economic burden for them (14). A review indicates that higher spending on cancer is consistently associated with lower mortality (15). In other words, loss of income and work-related benefits experienced by the unemployed may lead to adverse health outcomes (16). Unemployment of the family members further impairs the social support and financial source, resulting in unaffordability of the advanced therapies and, the progression of cancer and mental stress. This is supported by our finding that unemployment of family member was an independent risk factor for lower MS score. Additionally, the proportion of the use of immune therapy in melanoma patients with unemployment was only one-third of those who did not experience the unemployment. Considering the highly cost of immune therapy, which costs around 4000 USD monthly, the result indicates that financial status might be an essential consideration in the selection of treatment strategy. These findings indicate that the health disparities in cancer could result in a vicious circle. Social setting, incorporating poverty, culture, and social justice were found to play a part in disease outcome (17). Some poverty-related barriers such as income, education, and health insurance could influence melanoma outcomes (18). This makes social justice quite essential to diminish the disparities in melanoma.

To the best of our knowledge, this is the first empirical study of pandemic-related impacts on the HRQoL of melanoma patients. Our finding indicates that early and timely mental health intervention, telemedicine, and health education are needed for melanoma patients. More importantly, health decision makers should consider the reimbursement policy of advanced treatment methods for cancers with high fatality rate.

### Limitation

There are limitations in our study. First, we conducted an online survey instead of in-person interview because of the high infectivity of the COVID-19. Therefore, the representativeness of our sample might be limited owing to selection bias. Second, a total of 135 patients participated in the study despite our efforts to recruit as more patients as we could. The sample size is relatively small which may lead to insufficient power of test to identify differences. Third, the exposure and outcome variables were self-reported, and recall bias might be introduced. Last, the survey was conducted among Chinese patients, which may not fully represent melanoma patients beyond China owing to the differences in culture, reimbursement policy, and social system.

## Data Availability Statement

The raw data supporting the conclusions of this article will be made available by the authors, without undue reservation.

## Ethics Statement

The studies involving human participants were reviewed and approved by the institutional research ethics boards of Xiangya Hospital, Central South University (approval number: 202002024). The patients/participants provided their written informed consent to participate in this study.

## Author Contributions

XC and JS: conception and design. MS: administrative support. JS, SZ, MY, WB, and YW: provision of study materials or patients. YG, MS, and XZ: collection and assembly of data. YG, MS, and YX: data analysis and interpretation. All authors manuscript writing and final approval of manuscript.

## Conflict of Interest

The authors declare that the research was conducted in the absence of any commercial or financial relationships that could be construed as a potential conflict of interest.
