# Supplementary material for: Unemployment and Health-Related Quality of Life in Melanoma Patients During the COVID-19 Pandemic
Source: Front Public Health. 2021 Feb 22;9:630620. doi: 10.3389/fpubh.2021.630620 (PMC7937627; doi:10.3389/fpubh.2021.630620)
Supplement: Supplementary file 1 [file Table_1.DOC]

Q1. What is your biological sex? [Single-choice question]

Male

Female

Q2. What is your age (years)? [Fill in the blanks, numbers only]

Q3. What is your education level? [Single-choice question]

Primary school and below

Middle school

High school

College and above

Q4. What is your personal annual income? [Single-choice question]

<10,000

10,000–49,999

50,000–99,999

100,00–199,000

>200,000

Q5. What is your marital status? [Single-choice question]

Unmarried

Married

Widowed

Q6. What is the stage of melanoma according to the diagnosis of your doctor? [Single-choice question]

I

II

III

IV

Q7. How many years have you been diagnosed with melanoma? [Single-choice question]

<1 year

1–2 years

≥3 years

Q8. Where is your primary tumor? [Single-choice question]

Head/neck

Extremities

Trunk

Mucosa

Unknown

Others

Q9. Which treatment are you currently undergoing? [Multiple-choice question]

Chemotherapy

Radiotherapy

Immunotherapy

Targeted therapy

Others

Q10. What is your current status of melanoma? [Single-choice question]

Stable

Recurrent

Metastasis

Q11. Do you have other diseases? [Multiple-Choice Questions]

None

Hypertension

Diabetes

Coronary heart disease

Gout or hyperuricemia

Stroke (cerebral infarction, cerebral hemorrhage)

Respiratory system diseases (chronic bronchitis, tuberculosis, etc.)

Other [text description]

Q12. Have you been diagnosed with COVID-19? [Single-choice question]

Yes

No

Q13. During the past two weeks, how has your outdoor activity been affected? [single choice question]

My outdoor activity was unaffected

My outdoor activity was partly restricted

I was isolated at home and receiving medical observation

I was quarantined in hospital and receiving medical observation or treatment

Q14. What is your employment status since the epidemic of COVID-19? [Single-choice question]

I am unemployed since the epidemic

My employment status is unaffected since the epidemic

I was unemployed or retired before the epidemic

Q15. Are your family (family members who live together for more than 6 months during the past year) temporarily unemployed because of the epidemic? [Single-choice question]

Yes

No

Q16. Since the epidemic of COVID-19, is there any change in your monthly income? [Single-choice question]

Complete income loss

Income reduced

Income unaffected (including the situation that I had no income before the epidemic)

income increased

Q17. Below is a list of statements that other people with your illness have said are important**.** Please choose one number per line to indicate your response as it applies to the past 7 days.

| **PHYSICAL WELL-BEING** | **Not at all** | **A little bit** | **Some-what** | **Quite a bit** | **Very much** |
| --- | --- | --- | --- | --- | --- |
|
| I have a lack of energy | 0 | 1 | 2 | 3 | 4 |
| I have nausea | 0 | 1 | 2 | 3 | 4 |
| Because of my physical condition, I have trouble meeting the needs of my family | 0 | 1 | 2 | 3 | 4 |
| I have pain | 0 | 1 | 2 | 3 | 4 |
| I am bothered by side effects of treatment | 0 | 1 | 2 | 3 | 4 |
| I feel ill | 0 | 1 | 2 | 3 | 4 |
| I am forced to spend time in bed | 0 | 1 | 2 | 3 | 4 |
| **SOCIAL/FAMILY WELL-BEING** | **Not at all** | **A little bit** | **Some-what** | **Quite a bit** | **Very much** |
|
| I feel close to my friends | 0 | 1 | 2 | 3 | 4 |
| I get emotional support from my family | 0 | 1 | 2 | 3 | 4 |
| I get support from my friends | 0 | 1 | 2 | 3 | 4 |
| My family has accepted my illness | 0 | 1 | 2 | 3 | 4 |
| I am satisfied with family communication about my illness | 0 | 1 | 2 | 3 | 4 |
| I feel close to my partner (or the person who is my main support) | 0 | 1 | 2 | 3 | 4 |
| *Regardless of your current level of sexual activity, please answer the following question. If you prefer not to answer it, please mark this box and go to the next section.* |  |  |  |  |  |
| I am satisfied with my sex life | 0 | 1 | 2 | 3 | 4 |

Q18. Please choose one number per line to indicate your response as it applies to the past 7 days.

| **EMOTIONAL WELL-BEING** | **Not at all** | **A little bit** | **Some-what** | | **Quite a bit** | **Very much** |
| --- | --- | --- | --- | --- | --- | --- |
|
| I feel sad | 0 | 1 | 2 | | 3 | 4 |
| I am satisfied with how I am coping with my illness | 0 | 1 | 2 | | 3 | 4 |
| I am losing hope in the fight against my illness | 0 | 1 | 2 | | 3 | 4 |
| I feel nervous | 0 | 1 | 2 | | 3 | 4 |
| I worry about dying | 0 | 1 | 2 | | 3 | 4 |
| I worry that my condition will get worse | 0 | 1 | 2 | | 3 | 4 |
| **FUNCTIONAL WELL-BEING** | **Not at all** | **A little bit** | | **Some-what** | **Quitea bit** | **Very much** |
|
| I am able to work (include work at home) | 0 | 1 | | 2 | 3 | 4 |
| My work (include work at home) is fulfilling | 0 | 1 | | 2 | 3 | 4 |
| I am able to enjoy life | 0 | 1 | | 2 | 3 | 4 |
| I have accepted my illness | 0 | 1 | | 2 | 3 | 4 |
| I am sleeping well | 0 | 1 | | 2 | 3 | 4 |
| I am enjoying the things I usually do for fun | 0 | 1 | | 2 | 3 | 4 |
| I am content with the quality of my life right now | 0 | 1 | | 2 | 3 | 4 |

Q19. Please choose one number per line to indicate your response as it applies to the past 7 days.

| **ADDITIONAL CONCERNS** | **Not at all** | **A little bit** | **Some-what** | **Quite**  **a bit** | **Very much** |
| --- | --- | --- | --- | --- | --- |
|
| I have pain at my melanoma site or surgical site | 0 | 1 | 2 | 3 | 4 |
| I have noticed new changes in my skin (lumps, bumps, color(colour)) | 0 | 1 | 2 | 3 | 4 |
| I worry about the appearance of surgical scars | 0 | 1 | 2 | 3 | 4 |
| I have been short of breath | 0 | 1 | 2 | 3 | 4 |
| I have to limit my physical activity because of my condition | 0 | 1 | 2 | 3 | 4 |
| I get headaches | 0 | 1 | 2 | 3 | 4 |
| I have had fevers (episodes of high body temperature) | 0 | 1 | 2 | 3 | 4 |
| I have swelling or cramps in my stomach area | 0 | 1 | 2 | 3 | 4 |
| I have a good appetite | 0 | 1 | 2 | 3 | 4 |
| I have aches and pains in my bones | 0 | 1 | 2 | 3 | 4 |
| I have noticed blood in my stool | 0 | 1 | 2 | 3 | 4 |
| I have to limit my social activity because of my condition | 0 | 1 | 2 | 3 | 4 |
| I feel overwhelmed by my condition | 0 | 1 | 2 | 3 | 4 |
| I isolate myself from others because of my condition | 0 | 1 | 2 | 3 | 4 |
| I have difficulty thinking clearly (remembering, concentrating) | 0 | 1 | 2 | 3 | 4 |
| I feel fatigued | 0 | 1 | 2 | 3 | 4 |

Q20. Please circle or mark one number per line to indicate your response as it applies to the past 7 days.

| ***At the site of my melanoma surgery:*** | **Not at all** | **A little bit** | **Some-what** | **Quite**  **a bit** | **Very much** |
| --- | --- | --- | --- | --- | --- |
|
| I have swelling at my melanoma site | 0 | 1 | 2 | 3 | 4 |
| I have swelling as a result of surgery | 0 | 1 | 2 | 3 | 4 |
| I am bothered by the amount of swelling | 0 | 1 | 2 | 3 | 4 |
| Movement of my swollen area is painful | 0 | 1 | 2 | 3 | 4 |
| Swelling keeps me from doing the things I want to do | 0 | 1 | 2 | 3 | 4 |
| Swelling keeps me from wearing clothes or shoes I want to wear | 0 | 1 | 2 | 3 | 4 |
| I feel numbness at my surgical site | 0 | 1 | 2 | 3 | 4 |
| I have good range of movement in my arm or leg | 0 | 1 | 2 | 3 | 4 |

Q21. During the past two weeks, have you went to the hospital for treatment? [Single-choice question]

No

Yes, go to a medical and health institution

Q22. During the past two weeks, have you consulted with a doctor via phone, WeChat, or telemedicine platforms? [Single-choice question]

No

Yes

Q23. During the epidemic, are you adherent to treatment? [Single-choice question]

No treatment prescribed

Not adherent to the treatment

Adherent to the treatment

Q24. During the epidemic, what was the main reason that influence your treatment? [Multiple Choice Questions]

Forgot to take medication

Stop the drug due to severe reaction

Stop the medication after the symptoms improve

Restricted access to medicines

Unable to go to the hospital or clinic for injection or surgery

others
